# Supplementary material for: Identifying and characterizing SCRaMbLEd synthetic yeast using ReSCuES
Source: Nat Commun. 2018 May 22;9:1930. doi: 10.1038/s41467-017-00806-y (PMC5964233; doi:10.1038/s41467-017-00806-y)
Supplement: Supplementary file 1 — Supplementary Information [file 41467_2017_806_MOESM1_ESM.pdf]

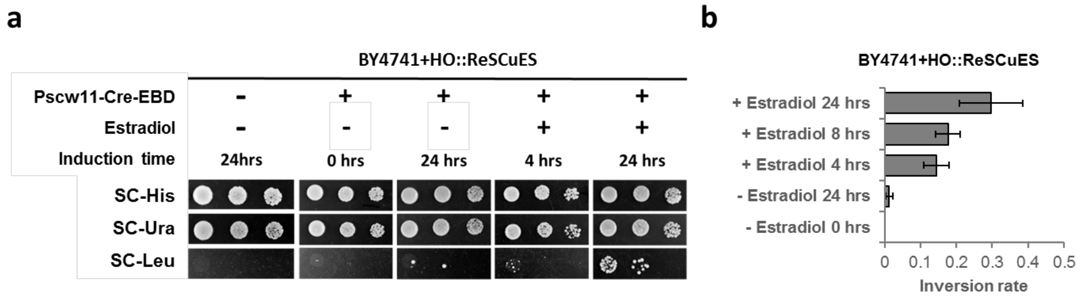

Supplementary Figure 1

Verification of ReSCuES in BY4741 background. (a) Cre-EBD generates Leu^+ phenotype in BY4741 strain with ReSCuES integrated at the *HO* locus. A single colony of indicated strains with or without pRS315-*Pscw11*-Cre-EBD plasmid were first overnight cultured in SC-His-Ura medium, and then diluted to OD_{600}=0.1 in SC-His medium, at which the time point was set as 0 hr. At each time point, the cells were collected and ten-fold serial dilution was conducted with a start concentration of 0.1 OD/ml. (b) Quantitative analysis the percentage of Leu^+ colonies in (a). Three independent colonies were analyzed for each condition.

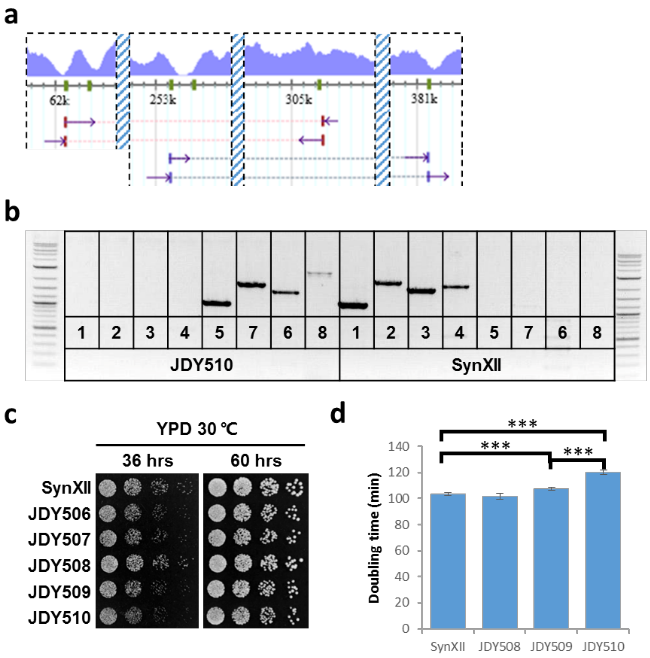

Supplementary Figure 2

Junction PCR to verify the 325,571 bp DNA rearrangement in JDY510. (a) The split read map indicated that this rearrangement was formed by two inversion events, which was cartooned in Fig. 2d. (b) Junction PCR analysis of JDY510. *synXII* genome was used as control. The amplicon were illustrated at Fig. 2d and their corresponding sizes are: 1=1,207 bp, 2=2,299 bp, 3=1,840 bp, 4=2,082 bp, 5=1,125 bp, 6=1,589 bp, 7=1,922bp, 8=2,792 bp. 1-4 were original junctions that could only be amplified using *synXII* genome. 5-8 were novel junctions that could only be amplified using JDY510 genome. (c) The growth of five SCRaMbLEd strains compared to that of *synXII* on YPD plate at two different time points. (d) The doubling time was measured for growth in YPD medium. *** is for a p-value <0.01 using 2 ends student t-test.

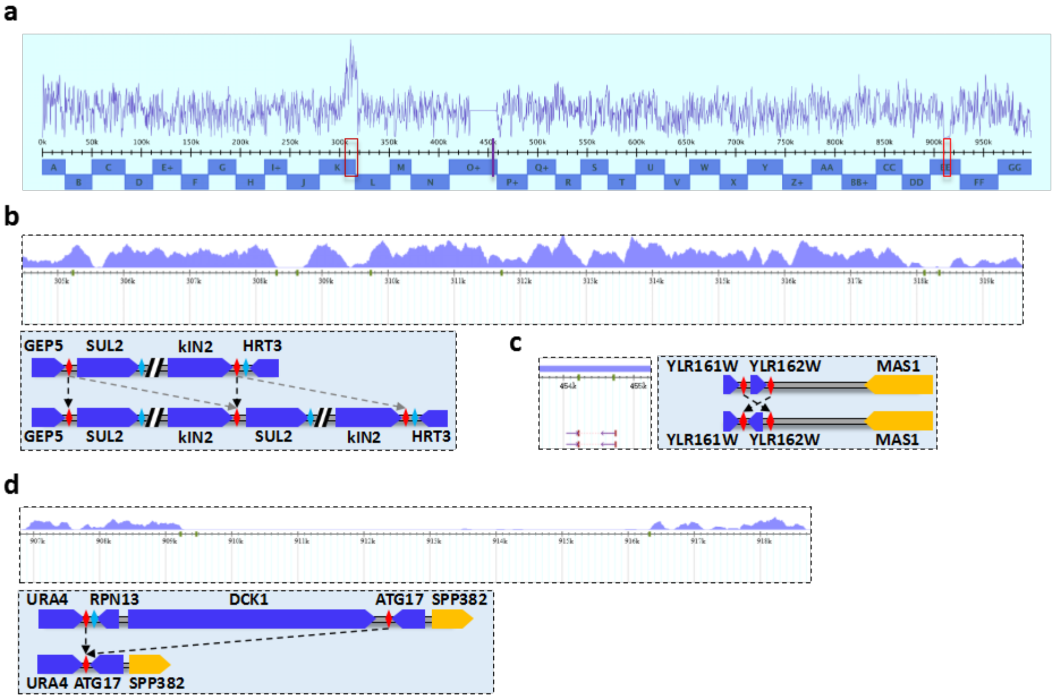

Supplementary Figure 3

***SynXII* rearrangements in JDY506.** (a) An overview of *synXII* was showed. Each synthetic megachunks was shown in blue and detected structural variations were marked by colored boxes. The sequencing reads were shown on the top and reads at the rDNA repeats were omitted due to the highly repetitive nature. A tandem duplication of 12,918 bp (b), an inversion of 531 bp (c) and a deletion of 7,098 bp (d) were identified in this strain. For each rearrangement, the split read map and a cartoon were showed to illustrate potential recombination events which produce the variation.

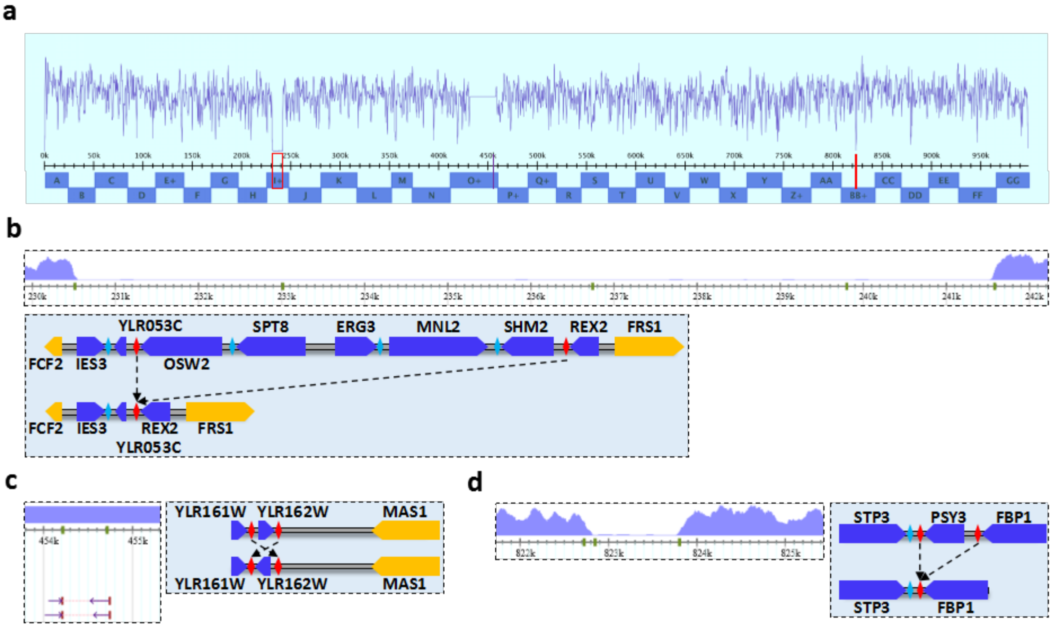

Supplementary Figure 4

***SynXII* rearrangements in JDY507.** The figure was presented the same as Supplementary Figure 3. (a) An overview of *synXII*. A deletion of 11,070 bp (b), an inversion of 531 bp (c) and a deletion of 954 bp (d) were identified in JDY507. The 531 bp inversion is the same as JDY506's.

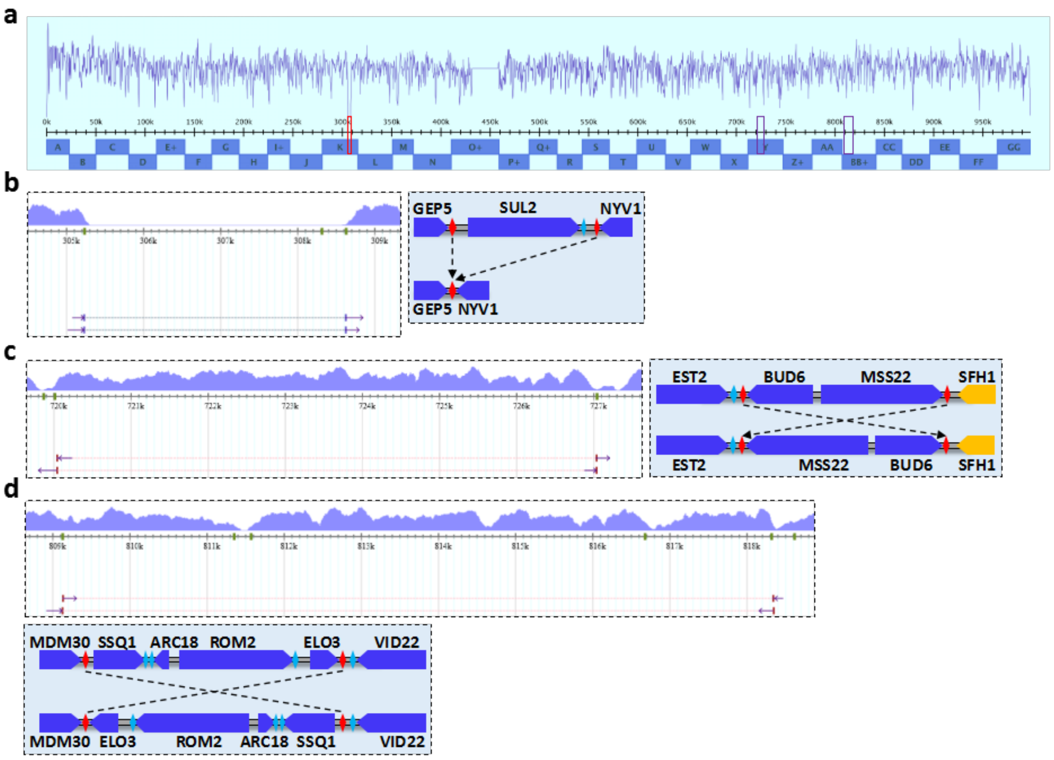

Supplementary Figure 5

***SynXII* rearrangements in JDY508.** The figure was presented the same as Supplementary Figure 3. (a) An overview of *synXII*. A deletion of 3,395 bp (b), an inversion of 7,062 bp (c) and an inversion of 9,225 bp (d) were identified in JDY508.

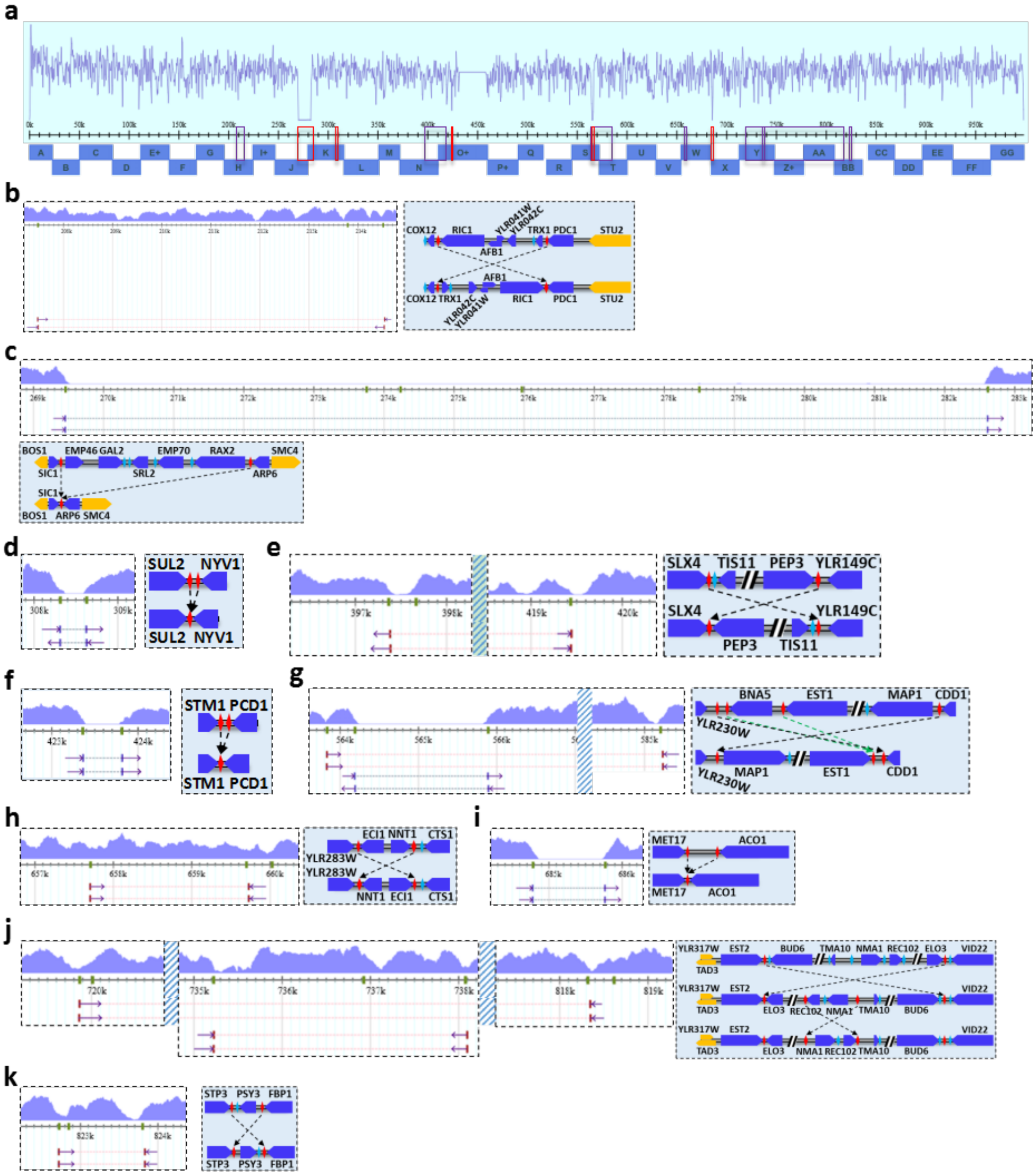

Supplementary Figure 6

***SynXII* rearrangements in JDY509.** The figure was presented the same as Supplementary Figure 3. (a) An overview of *synXII*. JDY509 contains the most rearranged structures, including five deletions and seven inversions: an inversion of 7,066 bp (b), a deletion of 13,161 bp (c), a deletion of 314 bp (d), an inversion of 22,104 bp (e), a deletion of 454 bp (f), a deletion of 1,701 bp overlaid with an inversion of 21,405 bp (g), an inversion of 2,023 bp (h), a deletion of 987 bp (i), an inversion of 1,736 bp overlaid with an inversion of 11. 98,488bp (j), and an inversion of 1,112 bp (k). Two regions (g and j) were found to involve more than one SCRaMbLE event.

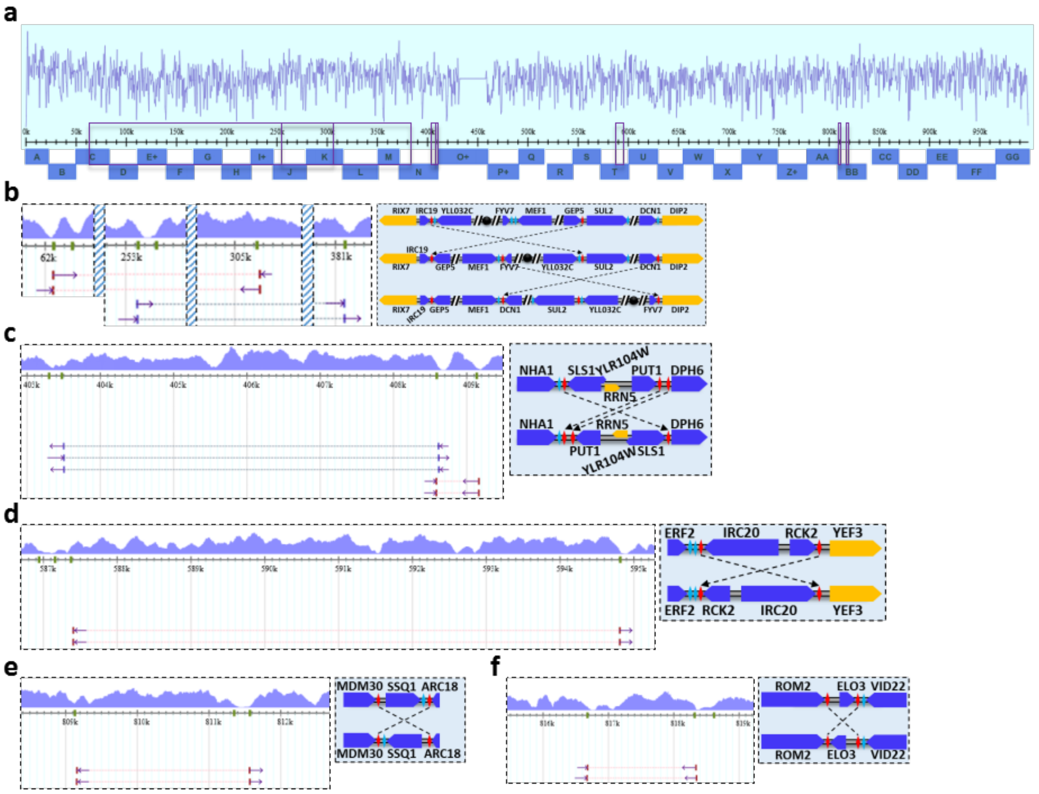

Supplementary Figure 7

***SynXII* rearrangements in JDY510.** The figure was presented the same as Supplementary Figure 3. (a) An overview of *synXII*. Only inversions were identified in JDY510 strain, including a superposed inversion of 325,571 bp (b), an inversion of 5,696 bp connected with another inversion of 4,580 bp (c), an inversion of 7,476 bp (d), an inversion of 2,476 bp (e) and an inversion of 1,675 bp (f).

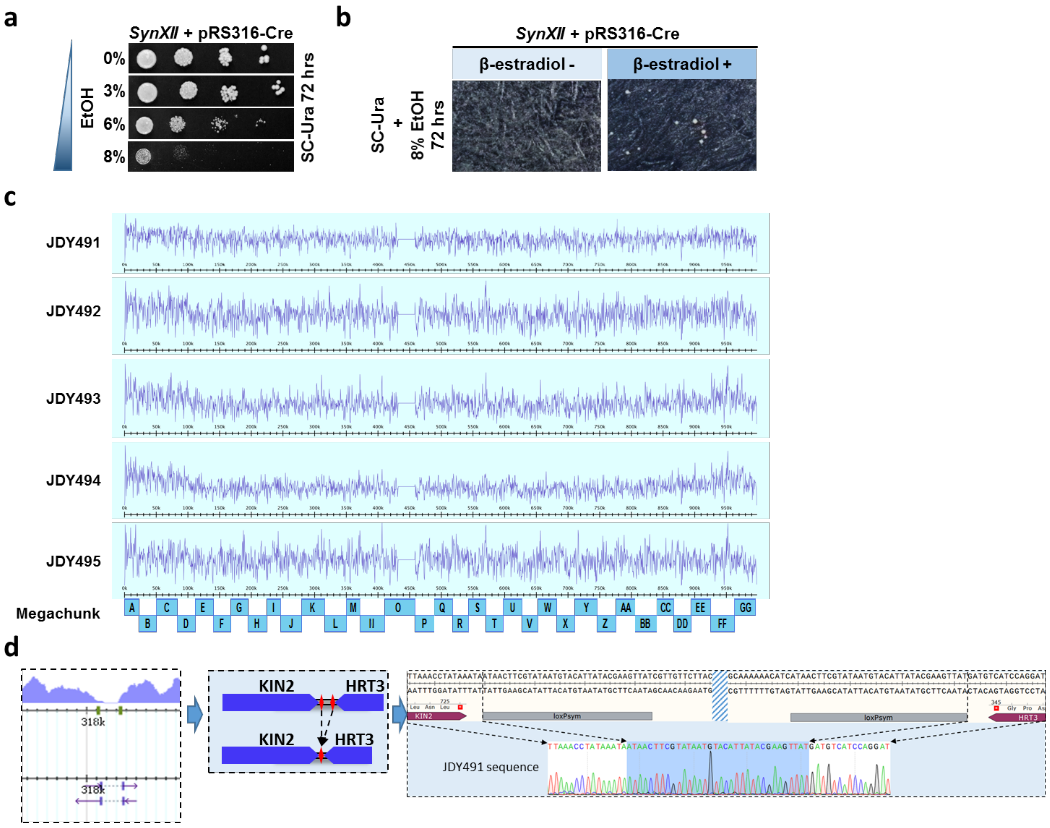

Supplementary Figure 8

Randomly isolated ethanol-resistant clones without ReSCuES are not necessary to be SCRaMbLEd. (a) Ethanol sensitivity test for *synXII* strain without ReSCuES and harboring a *URA3*-marked Cre plasmid (pRS316-Cre). Serial dilution (starting OD_{600}=1.0) was performed on SC-Ura medium containing different amount of ethanol. No estradiol was present. (b) *synXII* strain with or without inducing SCRaMbLE by estradiol was plated onto SC-Ura plates containing 8% of ethanol. The pictures were taken after incubating at 30°C for 72 hours. (c) Overview of sequencing reads of *synXII* in five randomly isolated ethanol-resistant clones (JDY491-JDY495). (d) Only a deletion of 224 bp between two adjacent *loxPsym* sites was identified in JDY491. The deletion was verified by the *Sanger* sequencing.

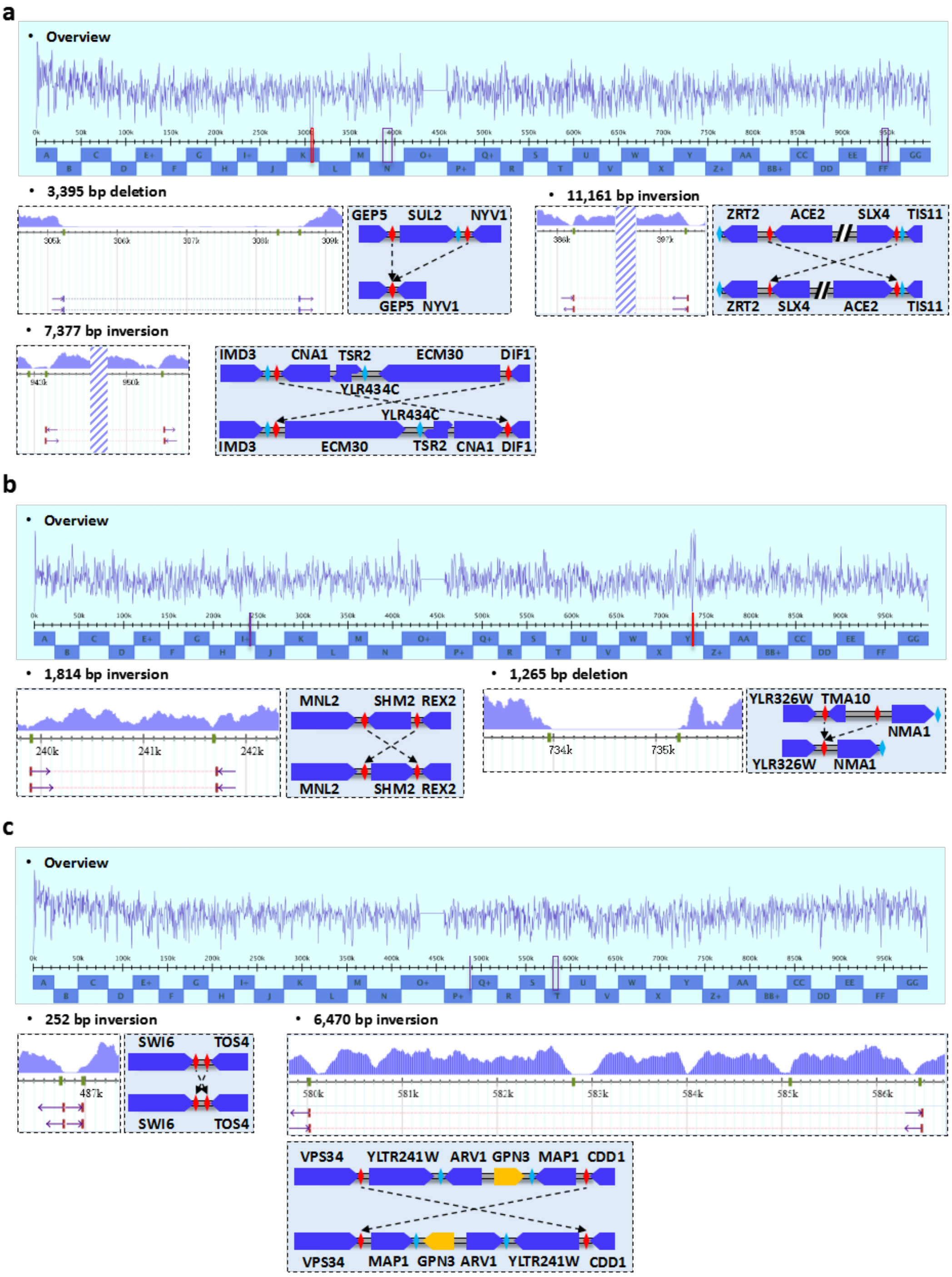

Supplementary Figure 9

Sequence rearrangement of *synXII* in three randomly isolated clones (JDY502, JDY504 and JDY505) by ReSCuES (Leu+ colonies). The figure was presented similar to that in Supplementary Figure 3. (a) *synXII* rearrangements in JDY502. A deletion of 3,395 bp, an inversion of 11,161 bp and an inversion of 7,377 bp were identified. (b) *synXII* rearrangements in JDY504. An inversion of 1,814 bp and a deletion of 1,265 bp were identified. (c) *synXII* rearrangements in JDY505. Two inversions, one of 252 bp and the other of 6,470 bp, were identified.

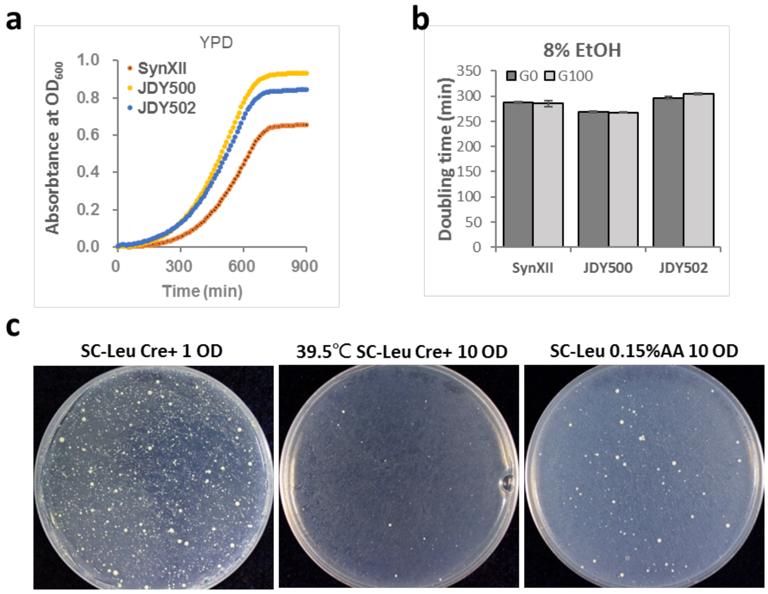

Supplementary Figure 10

Generate desired stress-tolerant strains by SCRaMbLE. (a) Growth curves of two selected ethanol-tolerant strains: JDY500 and JDY502 in YPD medium compared to that of the original *synXII* strain. The growth curves were measured using plate reader at 30°C with starting OD_{600}=0.1. (b) Doubling time of the strains. G0 is the original isolated clone and G100 is the same clone propagated for 100 generations. (c) Identification of SCRaMbLEd strains tolerant to high temperature and acetic acid. The *synXII* strain with ReSCuES was induced in β-estradiol for 4 hours before plating onto the selective medium or at the 39.5°C. Pictures were taken about incubating for 72 hours.

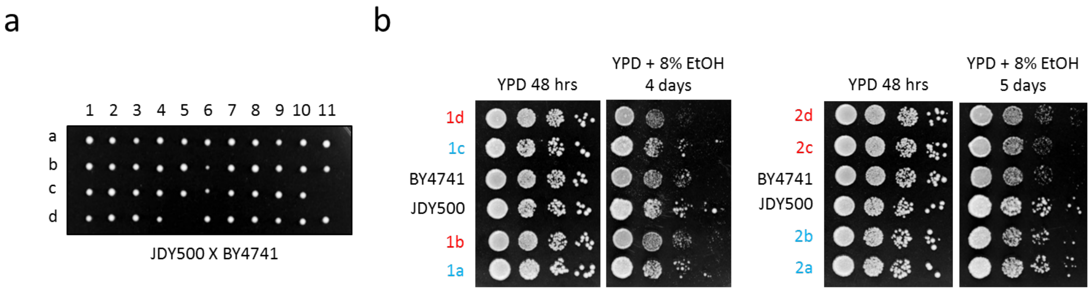

Supplementary Figure 11

Backcross and tetrad analysis of ethanol tolerant JDY500 strain. (a) Spore viability of JDY500 X BY4741 diploid. Ethanol JDY500 strain was backcrossed with BY4741, and then the diploid were sporulated and dissected onto YPD plate. (b) Ethanol sensitivity test for 4 spore tetrads, two examples (1a-1d and 2a-2d) were shown. Serial dilution (starting OD_{600}=1.0) was performed on YPD medium with or without 8% (V/V) ethanol. Based on the series dilution results, the spores in (a) could be divided into two groups: ethnaol tolerant group (blue label) and ethanol in-tolerant group (red label).

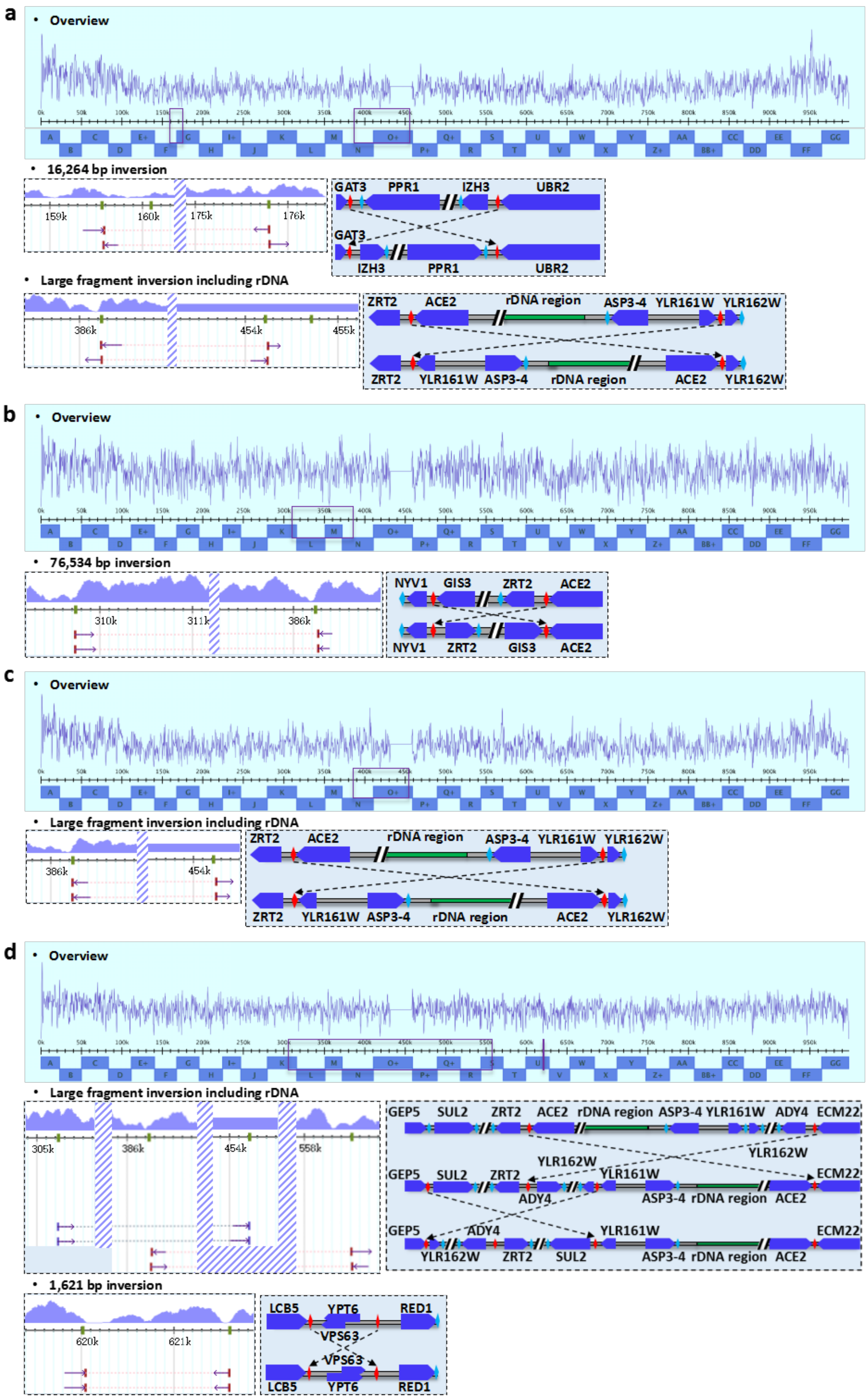
a
Overview
16,264 bp inversion
GAT3
PPR1
IZH3
UBR2
Large fragment inversion including rDNA
ZRT2
ACE2
rDNA region
ASP3-4
YLR161W
YLR162W
b
Overview
76,534 bp inversion
NYV1
GIS3
ZRT2
ACE2
c
Overview
Large fragment inversion including rDNA
d
Overview
Large fragment inversion including rDNA
GEP5
SUL2
ADY4
ECM22
1,621 bp inversion
LCB5
YPT6
VPS63
RED1

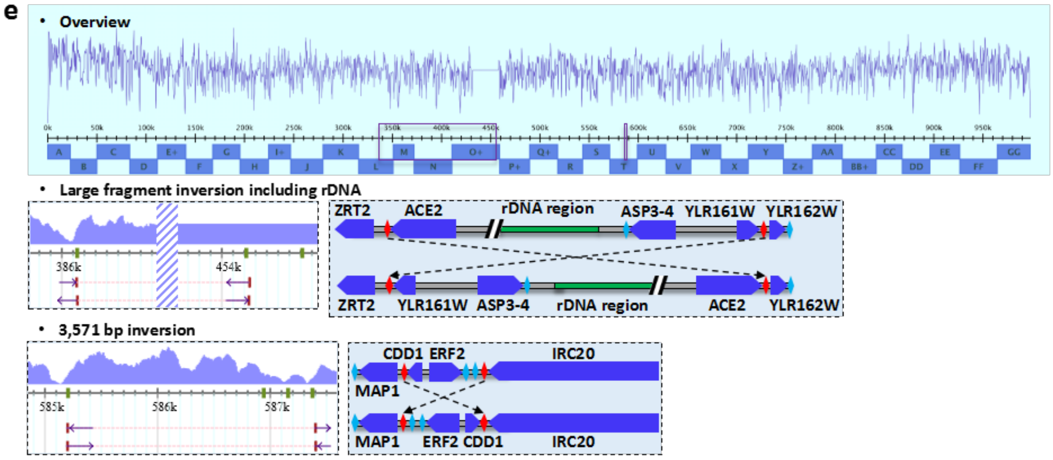

Supplementary Figure 12

Sequence rearrangements of *synXII* in five isolated clones (JDY496-500), which has similar phenotype to JDY500. The figure was presented similar to that in Supplementary Figure 3. (a) *synXII* rearrangements in JDY496, including two inversions. The second inversion happened between megachunk N and megachunk O changed the relative location of rDNA locus and led to the change of *ACE2's* 3'UTR. (b) *synXII* rearrangements in JDY497. The over 76 kb inversion changed *ACE2's* 3'UTR. (c) *synXII* rearrangements in JDY498. The inversion was the same as the second inversion of JDY496. (d) *synXII* rearrangements in JDY499, including two inversions. A very large superposed inversion from megachunk K to megachunk S and a small inversion in megachunk U were found. This large inversion also led to the change of *ACE2* 3'UTR. (e) *synXII* rearrangements in JDY500, including two inversions. A large inversion including rDNA locus and a small inversion in megachunk T were found. The large inversion is the same as what identified in JDY496 and JDY498. There are three *loxPsym* sites between ERF2 and IRC20, and the middle one is inserted as a land marker of deleting a tRNA gene.

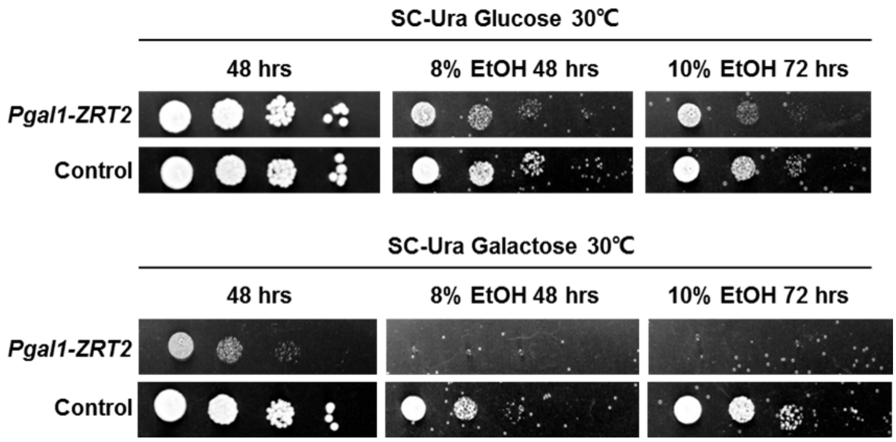

Supplementary Figure 13

Overexpression of *ZRT2* led to increased sensitivity to ethanol. 10-fold serial dilution (starting OD_{600}=1.0) was performed on SC-Ura medium containing 2% glucose or 2% galactose as carbon source. Without induction (SC-Ura glucose), the strain carrying *Pgal1-ZRT2* plasmid showed a slight ethanol sensitivity compared to the strain with empty control plasmid. Upon induction (SC-Ura galactose), overexpression of *ZRT2* caused severe growth defect even in medium without any ethanol and completely failed to grow when 8% or 10% ethanol was present.

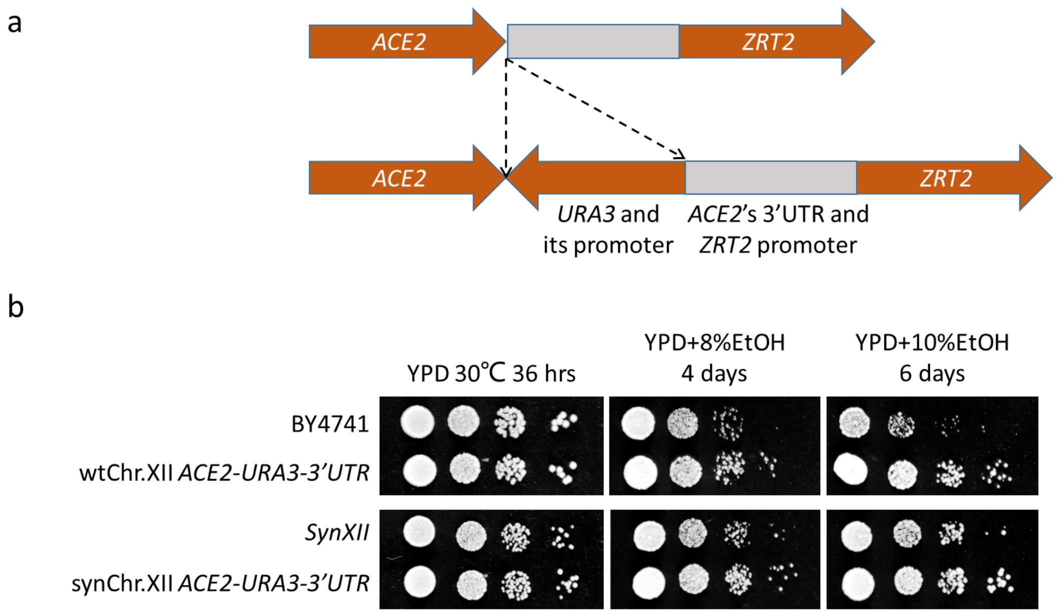

Supplementary Figure 14

Separating *ACE2* with its native 3'UTR could enhance the ethanol tolerance in both wild type and synthetic strain. (a) Schematic illustration of the strategy to separate *ACE2* coding sequence from the native 3'UTR by the *URA3* gene (b) Serial dilution assay to test the sensitive of strains in media containing different amount of ethanol. The cells were grew into log phase before they were collected, serial diluted and spotted onto the relative media.

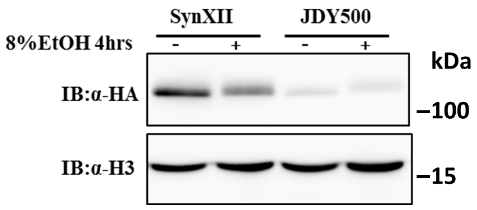

Supplementary Figure 15

Analyze the expression of Ace2p in JDY500 and *synXII* by tagging the endogenous Ace2p with 3HA. The native ACE2 promoter was used. Histone H3 was used as loading control. The log phase cells with or without ethanol treatment were collected and the whole cell proteins were extracted and subjected to western blotting.

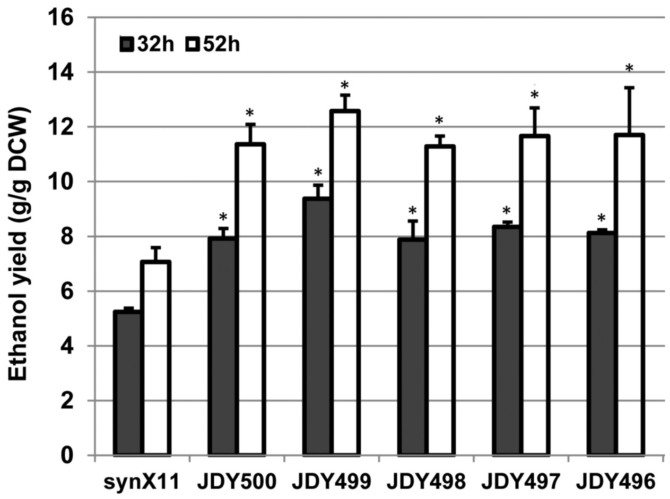

Supplementary Figure 16

Measurement of ethanol yield of SCRaMbLEd strains using static fermentation model. The asterisk (∗) indicates statistically significant differences (P< 0.01) between the parental *synXII* strain and SCRaMbLEd strains.

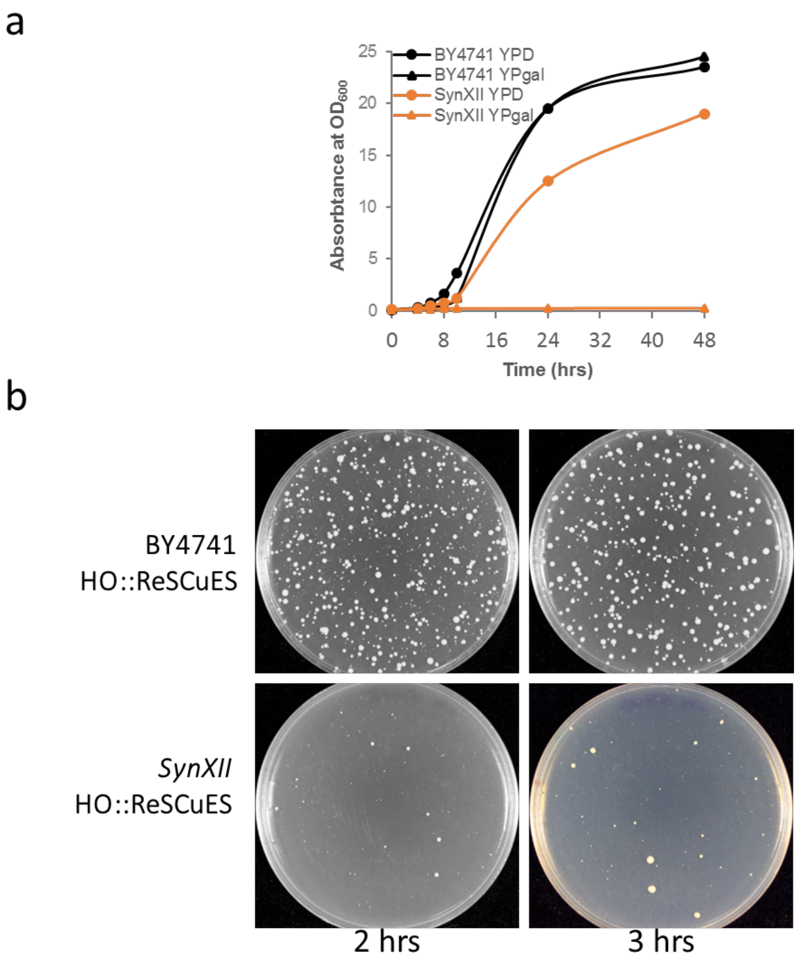

Supplementary Figure 17

Controlling Cre expression by Gals promoter causes significant loss of viability. (a) The growth curve of strains carrying the *Pgals*-NLS-Cre-EGFP plasmid with (YPgal) or without (YPD) induction. (b) Viability test for strains carrying the *Pgals*-NLS-Cre-EGFP plasmid after induction for 2 or 3 hours. 0.01 and 1.0 OD of cells were plated for BY4741 and *synXII* strain respectively. Pictures were taken after incubating the plates at 30°C for 48 hrs.

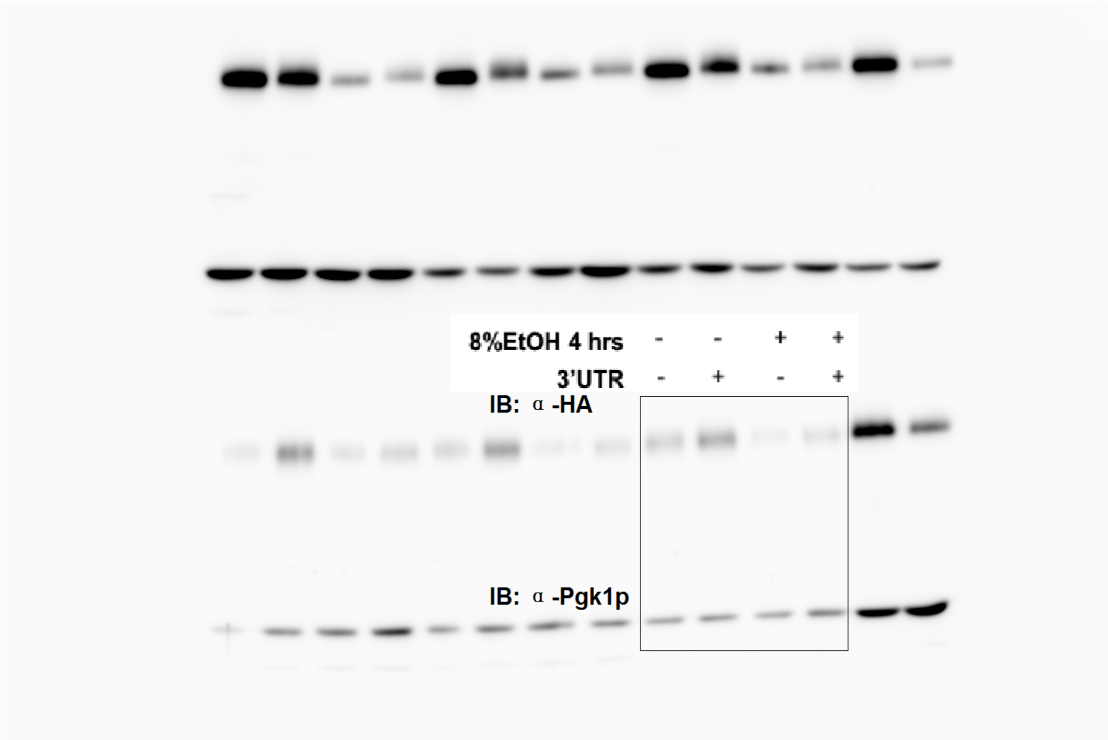

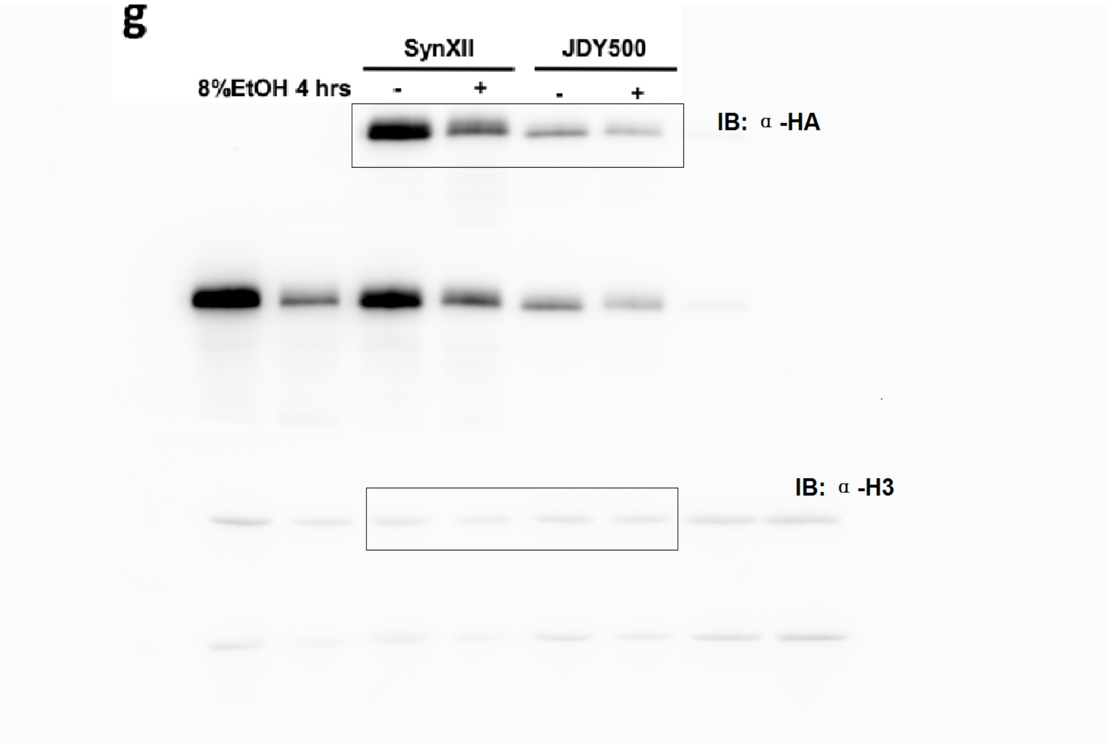

Supplementary Figure 18

The original blots for Figure 4f (upper panel) and 4g (bottom panel) before cropping.
