# Supplementary material for: Identifying and characterizing SCRaMbLEd synthetic yeast using ReSCuES
Source: Nat Commun. 2018 May 22;9:1930. doi: 10.1038/s41467-017-00806-y (PMC5964233; doi:10.1038/s41467-017-00806-y)
Supplement: Supplementary file 3 — Description of Additional Supplementary Files [file 41467_2017_806_MOESM3_ESM.pdf]

Description of Additional Supplementary Files

File Name: Supplementary Data 1
Description: PCR analysis of 91 chromosome XII loci

File Name: Supplementary Data 2
Description: Sequencing results summary and gene annotations for JDY506-JDY510

File Name: Supplementary Data 3
Description: Primers and plasmids used in this study

File Name: Supplementary Data 4
Description: Strains used in this paper
